# Oxidative Upgrading of Heavy Oil Residues with Polymer-Based Wastes for Sustainable Bitumen Production

**DOI:** 10.3390/polym17202747

**Published:** 2025-10-14

**Authors:** Yerbol Tileuberdi, Yerdos Ongarbayev, Aisulu Kabylbekova, Ernar Kanzharkan, Yerzhan Imanbayev, Ainur Zhambolova, Zhazira Mukatayeva, Nurgul Shadin

**Affiliations:** 1 Institute of Combustion Problems, 172, Bogenbai Batyr Str., Almaty 050012, Kazakhstan; erdos.ongarbaev@kaznu.edu.kz (Y.O.); erxank@mail.ru (E.K.); erzhan.imanbayev@mail.ru (Y.I.); zhambolova.ainur@mail.ru (A.Z.); jazira-1974@mail.ru (Z.M.); nugen_87@mail.ru (N.S.); 2 Institute of Natural Sciences and Geography, Abai Kazakh National Pedagogical University, Almaty 050010, Kazakhstan; 3 Faculty of Chemistry and Chemical Technology, Al-Farabi Kazakh National University, 71, Al-Farabi Ave., Almaty 050040, Kazakhstan; 4 Department of Chemistry and Chemical Technology, Faculty of Technology, M.Kh. Dulaty Taraz University, Taraz 080000, Kazakhstan

**Keywords:** bitumen, oxidation, heavy oil residues, polymer-containing waste, polyethylene, road construction

## Abstract

In this study, the oxidative upgrading of heavy oil residues using polymer-containing waste for the sustainable production of bitumen was investigated. Oxidation was performed at temperatures of 250–270 °C for 3–4 h with the addition of 2–3 wt.% polyethylene-based waste, under an air flow of 7 L/min. The physical and mechanical characterization of the resulting bitumen demonstrated compliance with oxidized modified bitumen grades OMB 100/130 and OMB 70/100. FTIR spectroscopy revealed the formation of carbonyl and sulfoxide functional groups, indicating the effective oxidative transformation of the bitumen matrix and partial incorporation of polyethylene fragments. NMR spectroscopy confirmed increased aromaticity and carbonyl content, while also detecting polyethylene-derived signals, suggesting compatibility and integration of the polymer waste into the oxidized structure. The thermal and rheological results showed that the optimal conditions for producing high-quality oxidized bitumen involved the use of 2% polymer waste at 270 °C for 4 h, yielding enhanced physical properties and chemical stability. These findings support the feasibility of using polymer-containing waste for bitumen upgrading, offering both environmental and technical advantages. The method not only improves the quality of bitumen but also contributes to waste valorization and circular economy practices in the road construction industry.

## 1. Introduction

In recent decades, the increasing volume of household waste containing polymer components has become one of the most pressing environmental problems. Plastics and other polymeric materials are highly resistant to decomposition, which leads to their accumulation in the environment and complicates their disposal. However, polymer waste is of considerable interest as a source of secondary raw materials for modifying building materials widely used in road construction and waterproofing, such as bitumen [1,2,3].

Bitumen is a complex organic mixture widely used in road construction and waterproofing systems, among other applications. According to statistics, 85% of all bitumen is used as a binder in several types of asphalt pavements, including those for roadways, airports, and parking lots; around 10% is used for roofing; and the remaining 5% is allocated for alternative applications, such as leak prevention, pipe asphalt, joint fillers, cable boxes, and sealing accumulators and batteries [4,5,6].

Several well-known manufacturing techniques can be used to make specification bitumen depending on the available raw materials and processing capacity. The following processes are frequently used: bitumen oxidation, solvent de-asphalting, vacuum distillation of thermally cracked residue, and distillation. Commonly referred to as blown bitumen, oxidized bitumen is produced at a facility called a bitumen-blowing unit, oxidizer, or air-blowing unit. The production of bitumen is reliant on the processing conditions and viscosity of the feedstock. The methods employ various chemical reactions that proceed to various extents to raise the bitumen’s average molecular weight and viscosity [6,7,8]. Bitumen production by oxidation is an irreversible process that results in an increase in the content of polar functional groups, increase in viscosity, and decrease in plasticity [9,10,11,12]. This process is characterized by a change in the softening temperature, decrease in penetration, and decrease in ductility [13,14]. Research shows that, upon exposure to oxygen in the oxidation method, asphaltenes are formed in the bitumen, making it more rigid and brittle [15].

However, during operation, the oxidative aging of bitumen occurs due to long-term exposure, and technogenic and high-temperature effects. These lead to rigidity, loss of elasticity, and cracking of the coating [12,15,16,17]. To solve this problem, methods for modifying bitumen to improve its rheological and mechanical properties are being actively studied [18,19,20,21,22]. One promising area is the use of polymer-containing waste, which can improve adhesion, increase heat resistance, and improve resistance to aging [23,24,25].

Despite a significant amount of research in the field of bitumen modification with polymers, the effect of polymer-containing household waste remains poorly understood. Most studies focus on commercial polymer additives, but household waste has high availability and environmental potential. When determining the prospects of new modifiers for vacuum residue oxidation, the main criteria are a high oxidation rate, energy efficiency, and rational resource consumption in terms of ensuring environmental safety. Therefore, from a scientific and practical point of view, it is important to study the effects of modifiers on the composition and properties of the products of the liquid-phase oxidation of vacuum residue [6].

The controlled oxidation of vacuum residue is an established industrial process for producing oxidized bitumen with improved characteristics. Unlike the oxidative aging that occurs during long-term pavement service and leads to undesirable embrittlement and cracking, intentional oxidation under regulated laboratory or industrial conditions allows the tailoring of the bitumen’s properties by adjusting the oxidation parameters and introducing functional additives [26]. This type of controlled process enables the enhancement of the heat resistance, stiffness, and elasticity of bitumen binders, especially when combined with polymeric waste as a modifying agent. Thus, the research aim is not to induce aging but to simulate and accelerate beneficial structural transformations in the binder matrix, resulting in improved final performance.

This study examines the effect of polymer-containing household waste on bitumen production through the oxidation of heavy oil residues in air blowing, aiming to obtain high-quality bitumen products by simplifying the technological parameters of the process. Particular attention is paid to the analysis of key physical and chemical characteristics, such as softening temperature, penetration, and ductility, which are the main indicators of its operational properties. This comprehensive study will allow us to evaluate the efficiency of using polymer waste to increase the durability of bitumen materials, which is of not only scientific but also practical importance for the construction and environmental industries.

## 2. Materials and Methods

### 2.1. Research Objects

Heavy oil residue (HOR) was used to obtain bitumen from “Asphaltobeton-1” LLP in Almaty (Kazakhstan). Heavy oil residue (also called vacuum residue) is a liquid with a density of 957.0 kg/m^3^ at 20 °C. The group and fractional compositions of the oil residues were preliminarily studied and are presented in Table 1.

As can be seen from Table 1, HOR contains large amounts of paraffin–naphthenic (25.9%) and heavy aromatic oils (18.1%). The total content of resins (31.5%) and asphaltenes (11.8%) is also significant. The fractional composition of the oil sludge, determined by distillation, was as follows (mass %): the initial boiling point was observed to be 180 °C; the fraction distilled between 200 °C and 350 °C accounted for 32.9 ± 0.2%, while the heavy fraction above 350 °C represented 60.1 ± 0.2%. The light fraction up to 200 °C constituted 7.0 ± 0.1%. Among the hydrocarbons, aromatic hydrocarbons (44.52%) and cycloalkanes (41.07%) were almost three times more abundant than alkane hydrocarbons (14.4%). Among the cycloalkanes, uncondensed cycloalkanes showed the highest content (17.24%). Among the aromatic hydrocarbons, naphthenobenzenes showed the maximum amount, equal to 7.91% [16].

The polyethylene waste used in this study was sourced from post-consumer packaging materials, specifically low-density polyethylene (LDPE) shopping bags (Kazakhstan Waste Recycling LLP, Almaty, Kazakhstan). Prior to use, the polyethylene pockets were visually inspected: they appeared as thin, transparent films with a slightly milky hue, typical of LDPE films. These materials were free from visible contamination. Before mixing with bitumen, the polyethylene bags were manually cut into small strips and then mechanically crushed using a multifunctional grinder (BioloMix, Dongguan, China) to obtain particles with a size of 1–2 cm. The crushed material retained its flexibility and showed no signs of thermal degradation, indicating suitability for thermal modification processes. To better understand the additive’s composition, a preliminary FTIR analysis of the polyethylene waste was carried out, confirming the characteristic absorption bands of LDPE (e.g., stretching vibrations of –CH_2_ at 2915 and 2848 cm^–1^, and rocking bands near 720 cm^–1^), with no detectable peaks of fillers or functional additives.

Table 2 presents the physical and mechanical properties of polymer waste.

The table shows that the tensile strength of the polymer waste was 8.9 MPa, while the initial plastic samples had a tensile strength of over 14 MPa. The relative elongation at break of the polymer waste was 210%, and the frost resistance was −45 °C.

**Figure 1:**
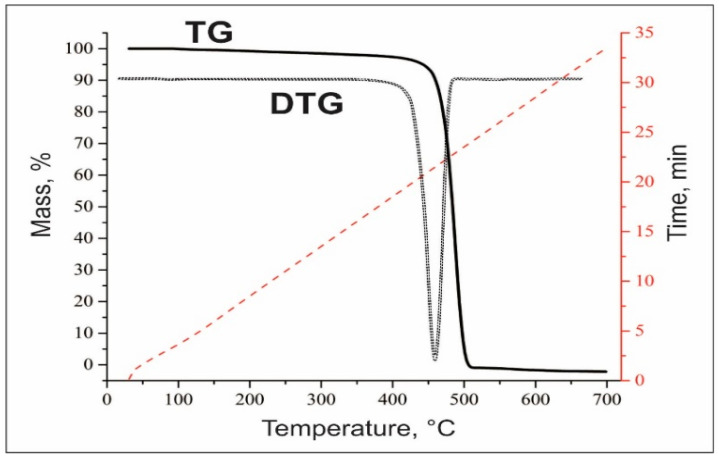
Thermogravimetric analysis of polymer waste.

Figure 1 shows the thermogravimetric (TG) analysis of polymer waste, according to which mass loss begins at 410 °C, and a sharp change is observed at 460 °C, which continues up to 510 °C. In the temperature range from 410 °C to 510 °C, the plastic waste lost 97 wt.%. The derivative thermogravimetry (DTG) curve shows that the chemical composition of the waste is single-component, or the content of other impurities is very small, because a distinct single peak is observed.

### 2.2. Methodology and Laboratory Setup for the Oxidation of Heavy Oil Residues with the Addition of Modifiers

**Figure 2:**
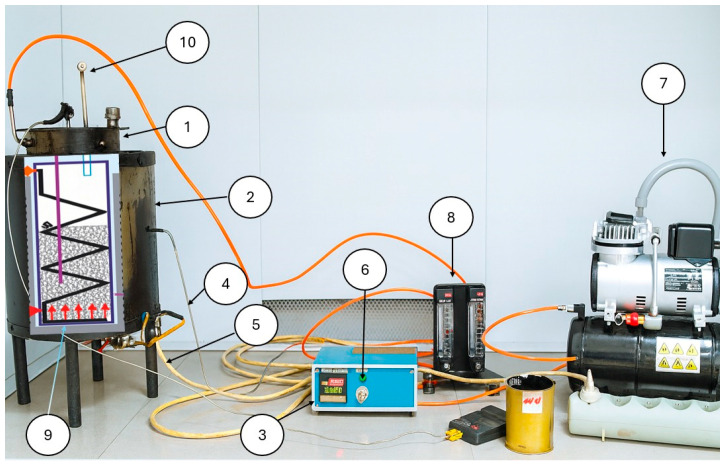
Schematic diagram of the heavy oil residue oxidation setup for bitumen production: 1—cylindrical reactor; 2—electric tubular furnace with vertical split; 3—thermocontroller with PID temperature regulator; 4—thermocouples (Type K, up to 1100 °C); 5—power supply; 6—temperature regulator; 7—compressor; 8—air flowmeter; 9—gas inlet nozzle; 10—exhaust gas flow.

The oxidative upgrading of heavy oil residues with polymer-based waste was carried out in a laboratory setup with the addition of modifiers, the scheme of which is shown in Figure 2. To obtain oxidized bitumen, 1.0–3.0 wt.% of polymer waste was added to the bitumen (Kazakhstan Waste Recycling LLP, Almaty, Kazakhstan), and oxidation was conducted at 250–270 °C for up to 4 h with air supplied at a rate of 7.0 L/min. The selected temperature regime ensured the effective involvement of polyethylene in the chemical structure of bitumen due to the formation of active functional groups necessary to increase the thermal stability and adhesive properties of the coating.

The setup consisted of a steel cylindrical reactor, which was heated electrically; the heating temperature was fixed with a thermostat. An air inlet nozzle was connected to the reactor. A stirrer was connected to the top of the reactor, which allowed for mechanical mixing of the raw material to intensify the process. The setup consisted of a batch reactor (1) LLP with a diameter of 159 mm and a capacity of 5 dm^3^. The reactor was heated in a cylindrical vertical furnace (2). Inside the reactor, there was a nozzle (9) for air supply, which was mounted in a spiral on the reactor walls, allowing the air for oxidation to enter in a heated state. The materials for the reactor and the air inlet nozzle were AISI 316 stainless steel, as this grade of stainless steel is resistant to high temperatures. The air flow rate was controlled using pre-calibrated flowmeters (Bes Saiman LLP, Almaty, Kazakhstan) (8). The process temperature was measured by thermocouples (Bes Saiman LLP, Almaty, Kazakhstan) located inside the reactor and the furnace (4).

### 2.3. Bitumen Characterization Methods

*Penetration*. Penetration is how far a calibrated needle (0.1 mm) penetrates into the volume of bitumen under the action of a fixed load for a specified time at a specified temperature. It was determined using the PNB-03 device (Spetsneftekhimavtomatika, Moscow, Russia) according to state standard (SS) 33136 [27]. Heated bitumen was poured into a special metal cup and left to harden at room temperature for 1 h. The glass was immersed in a water bath at +25 °C (if penetration is determined at 0 °C, the bath temperature should also be 0 °C) and left there for 1 hour. Then, the glass bath was placed on the movable table of the device, and the metal needle was positioned so that its tip touched the surface of the bitumen. Then, at metal needle loading *t_p_* = 5 s, the “start” button was pressed. The load was 100 g. In this case, the device automatically recorded the readings.

*Determination of softening temperature.* The softening temperature of the bitumen was determined in accordance with the requirements of state standard 33142 [28] using the “Ring and Ball” device on the LHDF-4 model (Spetsneftekhimavtomatika, Moscow, Russia). The bitumen was heated to a homogeneous state and poured into a metal ring, and after cooling it in air or at room temperature for 30 min, a convex surface was cut out with a cutter. Then, the sample was placed in a water bath heated at a rate of 5 °C per minute, after which a steel ball was placed on the bitumen layer in the flask. As the water temperature increased, the bitumen softened and the ball pressed on the bitumen and, under the action of gravity, passed through the ring. The softening temperature was determined by the “Ring and Ball” device when the ball touched the plate located under the ring.

*Ductility.* The ductility of the bitumen was determined using a DAF-1480 plastometer (Dorlab-Ltd, Moscow, Russia) in accordance with SS 33138 [29]. This indicator characterizes its ability to stretch into threads. Heated bitumen was poured into cylindrical metal molds and kept at room temperature for 1 h. Then, we smoothed the surface of the sample with a knife, after which we immersed the prepared sample in a water bath at +25 °C, in which it was kept for 1 h. We then placed it in a device that subjected the bitumen to a tensile load at +25 °C. The plasticity was determined by the length (cm) of the thread formed when it broke.

### 2.4. Methodology of FT-IR Spectroscopic Analysis

The measurements were carried out using a Shimadzu IRPrestige-21 FTIR spectrometer equipped with a Pike Technologies Miracle accessory based on the Attenuated Total Reflectance (ATR) technique (Shimadzu, Kyoto, Japan). The spectra were recorded in the range of 4000–400 cm^−1^ with a resolution of 4 cm^−1^, and each spectrum was averaged over 32 scans to improve the signal-to-noise ratio. The ATR crystal enabled direct analysis of the bitumen surface without requiring extensive sample preparation. This method allowed for the identification of functional groups and detection of oxidation products, such as carbonyl (C=O) and sulfoxide (S=O) groups, which are key indicators of bitumen aging.

### 2.5. Methodology of NMR Spectroscopic Analysis

The Nuclear Magnetic Resonance (NMR) spectra of the hydrogen (^1^H) and carbon (^13^C) nuclei of the samples were recorded on a JNM-ECA Jeol 400 spectrometer (JEOL Ltd., Kyoto, Japan) at 25 °C. The operating frequencies for the ^1^H and ^13^C nuclei were set to 399.78 MHz and 100.53 MHz, respectively. Samples weighing 60 mg were dissolved in 0.5 mL of deuterated chloroform (CDCl_3_) (Sigma Aldrich Rus LLC, Moscow, Russia). The chemical shifts of the ^1^H and ^13^C nuclei were measured relative to the signals of the residual protons or carbon atoms of the deuterated chloroform.

### 2.6. Methodology of Thermal Analysis

The thermal properties of the samples were studied by synchronous differential thermal analysis and thermogravimetry (DTA/TG) on a NETZSCH STA 449 F3 (NETZSCH-Geratebau GmbH, Selb, Germany) setup. The analysis was performed in a nitrogen atmosphere with a gas feed rate of 50 mL/min. Aluminum oxide crucibles were used. The samples were heated at a rate of 10 °C/min to 1000 °C. 

### 2.7. Methodology of Frequency Sweep Test

The frequency sweep was performed using a SmartPave 102e dynamic shear rheometer (Anton Paar, Graz, Austria) with a 25 mm wide parallel plate system set 1 mm apart. Tests were performed at five different temperatures—30 °C, 40 °C, 50 °C, 60 °C, and 70 °C—covering the typical service temperature range for bituminous binders. At each temperature point, the complex shear modulus (G*) and phase angle (δ) were measured over a logarithmically spaced frequency range from 0.1 to 100 rad/s. The strain amplitude was kept steady during testing at 0.1%, which is within the range where the material behaves predictably, making sure that the response remained linear viscoelastic. The experimental procedure followed the principles described in AASHTO T 315 (or EN 14770 for European standards) [30,31]. The frequency-dependent data were later shifted horizontally according to the time–temperature superposition principle and fitted using the Williams–Landel–Ferry (WLF) equation to construct the master curve of the binder.

## 3. Results and Discussion

### 3.1. Physical and Mechanical Characteristics of Bitumen Oxidation Products

Oxidation of heavy oil residues was performed at 250 °C to 270 °C without and with the addition of polymer waste in quantities from 1.0 to 3.0 wt.%. Certain time intervals (2, 3, and 4 h) were selected to test the oxidation product and its physical and mechanical parameters. The bitumen products that met the standard requirements are presented in Table 3. The indicators are compared with the requirements of the recommendations for producing oxidized modified bitumens (OMBs) according to the technical standard R RK 218-189-2022 [32].

As can be seen from the table, the products of bitumen oxidation at 250 °C for 4 h with the addition of 3% polymer-containing waste and at 270 °C for 4 h with the addition of 2% polymer waste met the physical and mechanical requirements of oxidized modified bitumen grade OMB 100/130. The physical and mechanical properties of the product of bitumen oxidation at 270 °C for 3 h with the addition of 3% polymer waste correspond to the oxidized modified bitumen grade OMB 70/100. Although bitumen’s aging in service is traditionally considered a factor in its deterioration, in this study, controlled HOR oxidation was purposefully induced for structural modification. The oxidation carried out with the participation of oxygen and polyethylene led to improved characteristics.

Thus, the optimal conditions for the oxidation of heavy oil residues in bitumens from the “Asphaltobeton-1” LLP with the addition of polymer waste were also determined: polymer waste content—2–3 wt.%; temperature—250–270 °C; oxidation time—3–4 h. The proposed technology for producing oxidized petroleum bitumens allows for both an increase in the quality of the bitumens obtained and a two-fold reduction in the process time. In the bitumen industry, heavy oil residue is usually oxidized for 8 h at 240–280 °C. This study has revealed the possibility of reducing the oxidation time to 3–4 h, which allows for reduced energy costs. It proves the competitiveness of the expected results.

Previous studies have reported longer oxidation times, ranging from 6 to 8 h, to achieve comparable changes in the properties of bitumen at similar temperatures (e.g., 240–260 °C), particularly in the absence of polymeric modifiers. For instance, Djimasbe et al. (2021) showed that extending oxidation to 6–7 h was necessary to achieve a softening point increase of ~10 °C [8]. The present study demonstrates that, with the addition of polyethylene-based modifiers, the desired viscosity and softening point can be achieved in a shorter period, contributing to energy efficiency and faster production. Comparative efficiency supports the industrial competitiveness of the developed process. These findings suggest that oxidation at elevated temperature significantly alters the structure and performance of the resulting bitumen.

Although the oxidation temperature of 250 °C exceeds the standard aging temperatures used in Rolling Thin-Film Oven Test (RTFO, 165 °C) and Pressure Aging Vessel (PAV, 100 °C) methods [10,12], this condition was intentionally applied to simulate the industrial air-blowing process of bitumen production. At this temperature, not only oxidative aging but also secondary reactions such as volatilization, mild pyrolysis, and polymerization/condensation of heavy hydrocarbons may occur. These transformations lead to structural changes in the colloidal system of the bitumen, increasing its rigidity and softening point, and altering its viscoelastic behavior. Phase separation can also become more pronounced at higher modifier concentrations. These phenomena were considered in the interpretation of the test results, and the polymer additive was expected to reduce the negative impact of such high-temperature aging by acting as a physical stabilizer and increasing the thermal resistance of the final product.

### 3.2. FT-IR Spectroscopic Analysis of Bitumen Samples

Fourier Transform Infrared (FTIR) spectroscopy was employed to assess the chemical structure and degree of oxidation of bitumen samples modified with polymer-containing household waste. The samples were prepared by oxidizing bitumen (oil residue from “Asphaltobeton-1” LLP) at temperatures ranging from 250 to 270 °C for 3 to 4 h with 2–3 wt.% of polyethylene-based waste.

**Figure 3:**
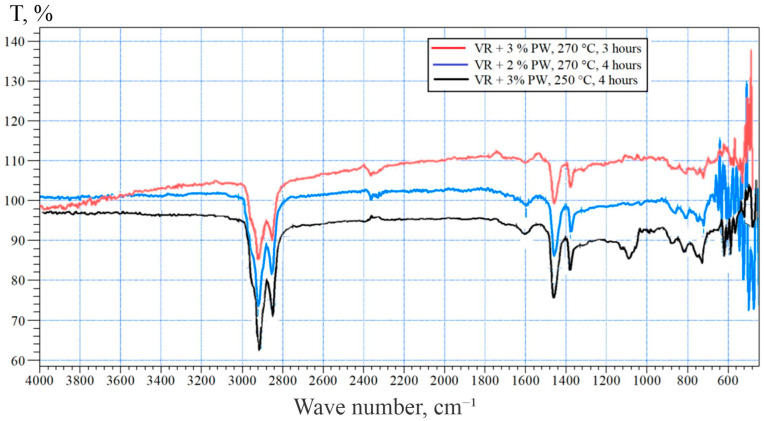
FTIR spectra of bitumen oxidation products with the addition of polymer-containing waste.

These findings are further supported by the FTIR spectra presented in Figure 3, which illustrates the characteristic absorption bands of the oxidized bitumen samples. The spectra clearly show variations in the intensity of the carbonyl (~1700 cm^−1^) and sulfoxide (~1030 cm^−1^) bands, as well as the preserved aliphatic C–H stretching regions (~2920 and 2850 cm^−1^), reflecting the degree of oxidation and the presence of polyethylene fragments.

The obtained FTIR spectra (Figure 3) provides valuable insights into the chemical transformations occurring in the bitumen matrix under the influence of polyethylene-containing waste and oxidative treatment. All the analyzed samples exhibit prominent absorption bands near 2920 cm^−1^ and 2850 cm^−1^, corresponding to the asymmetric and symmetric stretching vibrations of methylene (–CH_2_–) groups, which are characteristic both for the hydrocarbon skeleton of bitumen and for polyethylene fragments embedded in the matrix. Additional signals at ~1455 cm^−1^ and ~1375 cm^−1^ are assigned to bending vibrations of CH_2_ and CH_3_ groups, further supporting the aliphatic nature of the samples and the incorporation of polyolefin residues.

A notable feature in the spectra is the emergence of absorption bands in the 1700–1710 cm^−1^ region, which are attributed to the C=O stretching vibrations of carbonyl-containing species such as ketones, aldehydes, and carboxylic acids. The increase in the intensity of these bands in samples subjected to air oxidation indicates a higher degree of oxidative degradation. This trend directly correlates with the oxygen supply during processing, suggesting that oxidative pathways are enhanced in the presence of polyethylene and active air flow, promoting the formation of oxygenated species.

Furthermore, the bands in the 1030–1040 cm^−1^ region correspond to S=O stretching vibrations of sulfoxide groups, which are widely recognized as secondary oxidation markers in bitumen aging studies. The intensity of these bands also varies with processing conditions, supporting the hypothesis of intensified oxidation in samples exposed to air flow.

Overall, the FTIR data demonstrates that the incorporation of polyethylene-containing waste, particularly under oxidative conditions, significantly alters the chemical structure of bitumen by introducing oxygen-containing functionalities and retaining polymer-derived aliphatic structures.

Comparative analysis of samples revealed the following:

- Sample 1 (B-3-250-4), produced at 250 °C for 4 h with 3% polymer, exhibited moderate carbonyl and sulfoxide bands, suggesting balanced oxidation. The well-defined aliphatic peaks imply successful integration of the polyethylene waste.
- Sample 2 (B-2-270-4), obtained at 270 °C for 4 h with 2% polymer, showed a similarly strong carbonyl peak with slightly lower sulfoxide intensity, consistent with OMB 100/130 standards [32].
- Sample 3 (B-3-270-3), oxidized at 270 °C for 3 h with 3% polymer, demonstrated stronger carbonyl and sulfoxide absorption, indicating a higher degree of oxidation. This is consistent with the physical properties corresponding to OMB 70/100 grade.

These findings confirm that the addition of polyethylene-containing waste enhances the oxidative behavior of bitumen and contributes to the formation of structurally modified products. The FTIR method proved to be effective for monitoring chemical changes and assessing the compatibility of polymer additives with oxidized bitumen.

In summary, the FTIR results confirm that both temperature and polymer content significantly influence the oxidative behavior and final structure of the modified bitumen. Higher temperatures and polymer dosages promote deeper oxidation and functional group formation without impairing structural stability due to the compatibility of polyethylene waste with the bitumen matrix.

### 3.3. Nuclear Magnetic Resonance Spectroscopy Analysis

**Figure 4:**
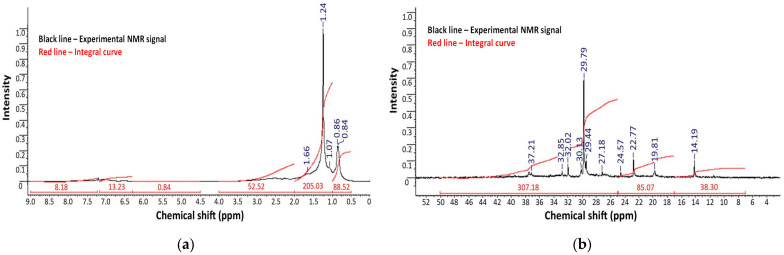
(**a**) ^1^H and (**b**) ^13^C NMR spectra of oxidized bitumen at 250 °C.

**Figure 5:**
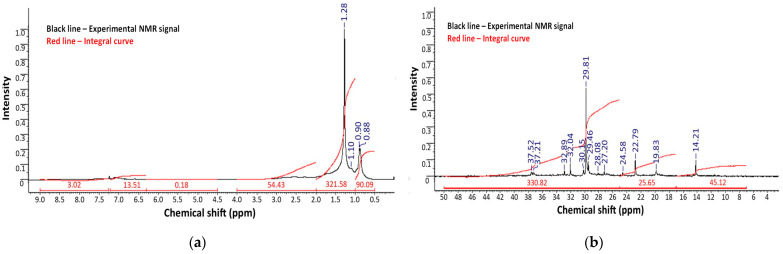
(**a**) ^1^H and (**b**) ^13^C NMR spectra of oxidized bitumen at 270 °C.

Nuclear Magnetic Resonance (NMR) spectroscopy was performed to characterize the molecular structure and compositional differences of the vacuum residues, polyethylene, and oxidized bitumen samples modified with polymer and rubber additives. Both proton (^1^H) and carbon (^13^C) NMR spectra were analyzed (Figure 4 and Figure 5), supplemented by two-dimensional (2D) techniques for polyethylene. Because the bitumen samples B-3-250-4 and B-2-270-4 were obtained by oxidation at two different temperatures, their characteristics correspond to the requirements of the same standard, OMB 100/130; they were compared and studied.

The comparative results of the NMR analysis are summarized in Table 4, which highlights the key observations for each sample and the impact of oxidation conditions and polymer content on the chemical composition of the bitumen.

For the raw vacuum residues from the “Asphaltobeton-1” LLP, the ^1^H and ^13^C NMR spectra exhibit typical signals of highly aliphatic hydrocarbons with broad, poorly resolved resonances, indicating the complex, polydisperse nature of bitumen. The ^1^H spectra are dominated by methylene (–CH_2_–) and methyl (–CH_3_) groups, observed in the 0.8–2.0 ppm range, with minor signals corresponding to aromatic protons between 6.5 and 8.0 ppm. The ^13^C spectra confirm this, with strong aliphatic carbon signals (10–50 ppm) and weaker, broad signals in the aromatic region (120–150 ppm).

Upon oxidation, the chemical structure of bitumen undergoes significant transformation, as evidenced by changes in the NMR profiles (Figure 4 and Figure 5). The ^1^H NMR spectra of oxidized samples reveal an increase in aromatic proton intensity (6.5–8.0 ppm), consistent with aromatic condensation reactions and asphaltene formation. In addition, new downfield signals at ~9–10 ppm emerge, corresponding to aldehyde or carboxylic acid protons, indicating oxidative degradation. These changes are further supported by the ^13^C spectra, where the appearance of peaks in the 170–200 ppm region confirms the formation of carbonyl-containing compounds, such as ketones, aldehydes, and carboxylic acids—hallmarks of oxidative aging.

A comparison of oxidation at two temperatures (250 °C and 270 °C) reveals that the higher temperature promotes deeper oxidation. The sample treated at 270 °C displays more intense signals in both the aromatic and carbonyl regions, suggesting enhanced molecular condensation and oxygen uptake. Simultaneously, the intensity of aliphatic signals diminishes, reflecting the degradation of saturated hydrocarbon chains and the incorporation of oxygenated species into the bitumen structure.

These observations align with previous studies on the thermal–oxidative aging of bitumen, where increased temperature correlates with the enrichment of aromatic and polar functionalities. Thus, NMR spectroscopy confirms that polyethylene-modified bitumen, when subjected to air oxidation, undergoes a transformation toward a more aromatic and oxygenated matrix, which is critical for understanding changes in its performance characteristics.

Overall, the NMR data demonstrates the progressive chemical transformation of vacuum residue during oxidation in the presence of polymer waste. Polyethylene signals are still visible for the modified bitumen, suggesting polyethylene’s incorporation into the bituminous matrix. Moreover, the emergence of new aromatic and oxygenated groups, as shown in the ^13^C and ^1^H NMR spectra, indicates ongoing oxidative polymerization and functional group transformation. Compared to the unmodified sample, oxidized samples exhibited increased aromaticity and reduced aliphatic content, supporting the hypothesis of enhanced structural network formation. These observations provide molecular-level evidence of the stabilizing role of polymer waste in the oxidation process.

### 3.4. Analysis of Thermal Properties of the Bitumen Samples

**Figure 6:**
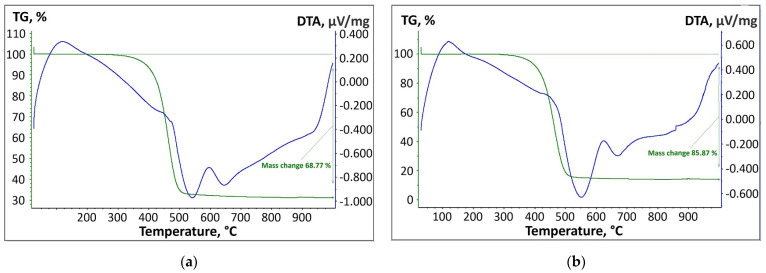
Thermograms of the bitumen samples based on the results of differential thermal analysis and thermogravimetry: (**a**) B-3-250-4 and (**b**) B-2-270-4.

The thermal properties of the bitumen samples B-3-250-4 and B-2-270-4 were studied by differential thermal analysis and thermogravimetry (Figure 6). According to the thermogravimetric curves (green lines), the masses of both samples do not change up to 350 °C. Then, in the temperature range above 500 °C, there was an intensive mass loss. The range of 350–500 °C was the main thermal destruction zone, during which the medium-molecular-weight fatty fractions contained in the bitumen evaporated and decomposed. Thermal decomposition of asphaltenes and resins occurred. In the B-3-250-4 sample, during thermolysis until 1000 °C, the total mass loss was 68.77 % (Figure 6a). The B-2-270-4 sample showed a mass loss of 85.87% before the thermolysis process (Figure 6b).

There is no significant difference in the derivative thermogravimetry curve between the two bitumen samples. In the range of 20–150 °C, an endothermic peak was observed in the DTA as a result of the evaporation of light hydrocarbons and water contained in the bitumen. Two endothermic minimums were observed in the DTA in the region of 550 °C and 650 °C. This is explained by the main thermal destruction of asphaltenes and resins, as well as the complete combustion of residual carbon. However, in the range of 600–650 °C, a weak exothermic peak was observed due to the slow oxidation of carbon residues. As the temperature increases above 650 °C, a strong exothermic effect is recorded, because, at this time, the residual carbon can be completely burned.

### 3.5. Rheological Characteristics of the Bitumen Samples

**Figure 7:**
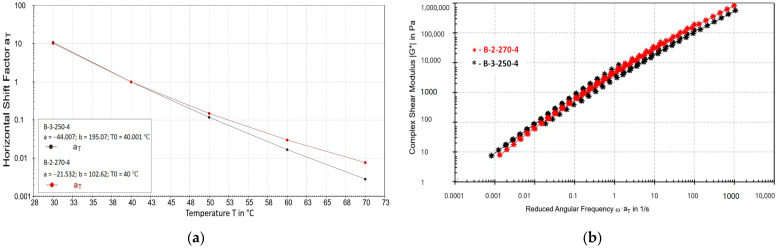
Master curves of the bituminous compositions modified with elastomeric additives: (**a**) Temperature dependence of the horizontal shear coefficient and (**b**) Master curves of the complex shear modulus.

The rheological performance of bituminous compositions was evaluated by frequency sweep tests, and the corresponding master curves of the complex shear modulus (|G*|) and temperature shift factors were constructed using the Williams–Landel–Ferry (WLF) model as shown in Figure 7.

Both curves of bitumen samples B-3-250-4 and B-2-270-4 demonstrate a tendency for a logarithmic decrease in the horizontal shear coefficient (aT) with increasing temperature. The curve in Figure 7a corresponding to sample B-2-270-4 (red line) is located higher than the curve of sample B-3-250-4 (black line) throughout the studied interval. This fact indicates that a higher shear coefficient is required to match the experimental data of sample B-2-270-4, which indicates that it has lower temperature sensitivity compared to sample B-3-250-4. The greatest discrepancy is observed at low temperatures (30–40 °C), whereas at higher temperatures (around 70 °C), the differences become less pronounced. Consequently, sample B-2-270-4 demonstrates higher temperature stability and a greater ability to maintain structural integrity across a wider temperature range.

For both studied samples, master curves of the complex shear modulus |G*| (in Figure 7b) were constructed depending on the reduced angular frequency (ω aT). The obtained dependences have a similar shape and a close slope angle, which indicates the same nature of relaxation processes in the materials. At the same time, it can be noted that the curve for sample B-2-270-4 (red line) is located slightly higher compared to the curve of sample B-3-250-4 (black line) through almost the entire range of studied frequencies. The difference indicates that sample B-2-270-4 has a higher complex modulus |G*|, which reflects its greater rigidity and resistance to shear deformations. This distinction is especially pronounced in the medium-frequency range (approximately 0.1–10 rad/s), which corresponds to operational loads typically experienced by asphalt pavements. 

These findings provide important insights into the performance of modified bitumen under real service conditions. The enhanced rigidity and reduced temperature sensitivity of sample B-2-270-4 suggest superior suitability for pavements exposed to fluctuating climatic conditions and heavy traffic. Such rheological improvements are expected to contribute to extended service life, reduced maintenance costs, and improved sustainability of asphalt pavements. Thus, while both bitumens produced by the oxidation of heavy oil residues with the polymer-containing wastes demonstrate improved rheological properties, sample B-2-270-4 clearly exhibits a more advantageous balance of rigidity and temperature stability compared to sample B-3-250-4.

## 4. Conclusions

The addition of polymer waste during the oxidation of heavy oil residues enhances bitumen quality, offering an eco-friendly approach for sustainable road materials and promoting a circular economy in construction.

- The study confirmed that incorporating 2–3 wt.% polymer-based waste in the oxidation of heavy oil residues significantly improves the physical and mechanical properties of the resulting bitumen.
- The optimal oxidation conditions were identified as 270 °C for 4 h with 2% polymer waste, resulting in products that meet the OMB 100/130 grade requirements according to RP RK 218-189-2022.
- FTIR spectroscopy revealed key functional groups formed during oxidation, such as carbonyl and sulfoxide bands, which correlate with enhanced oxidative aging and structural modification of the bitumen.
- NMR spectroscopy further demonstrated chemical transformations, including increased aromaticity and the appearance of oxygenated functionalities, confirming the deeper oxidation and successful integration of polyethylene fragments.
- Thermal analyses demonstrated that both bitumen samples experienced stability up to 350 °C, followed by significant decomposition of resins and asphaltenes, with distinctive carbon oxidation behavior above 600 °C.
- Rheological evaluation confirmed that both oxidized polymer-modified bitumens exhibit enhanced performance; however, B-2-270-4 demonstrated superior rigidity, reduced temperature sensitivity, and greater structural stability across the operational frequency range studied.

The proposed oxidative upgrading strategy provides an environmentally friendly solution for utilizing plastic waste while enhancing bitumen quality, aligning sustainable construction and circular economy principles.

## Figures and Tables

**Table 1:** Group and fractional compositions of heavy oil residue [6].

Indicator	Heavy Oil Residue
Content of the oil fractions, mass. %:	
Paraffinic–naphthenic	25.9 ± 0.2
Light aromatic	9.9 ± 0.1
Medium aromatic	2.8 ± 0.1
Heavy aromatic	18.1 ± 0.1
Content of resin fractions, mass. %:	
Neutral resins	11.2 ± 0.1
Acidic resins	20.3 ± 0.2
Content of asphaltenes, mass. %	11.8 ± 0.1
Fractional compositions, mass. %:	
The beginning of boiling—180 °C	7.0 ± 0.1
200–350 °C	32.9 ± 0.2
350 °C—end of boiling	60.1 ± 0.2

**Table 2:** Physical and mechanical properties of polymer waste.

Indicator	Result
Tensile strength, MPa	8.9
Relative elongation at break, %	210
Frost resistance, °C	−45

**Table 3:** Physical and mechanical characteristics of bitumen oxidation products with the addition of polymer-containing waste.

**Physical and Mechanical Properties**	**Modifier Content, Oxidation Temperature, Oxidation Time, and Requirements**					**Normative Document on Test Methods**
	3%, 250 °C, 4 h	2 %, 270 °C, 4 h	Requirements for OMB 100/130	3 %, 270 °C, 3 h	Requirements for OMB 70/100	
Sample Name	B-3-250-4	B-2-270-4		B-3-270-3		
Penetration at 25 °C, 0.1 mm	117	128	101–130	100	71–100	GOST 33136-2014 [27]
Softening point, °C	49	48	48	54	52	GOST 33142-2014 [28]
Ductility at 25 °C, cm	23	39	14	30	12	GOST 33138-2014 [29]
Bitumen grade according to P RK 218-189-2022	OMB 100/130			OMB 70/100		-

**Table 4:** Comparative interpretation of ^1^H and ^13^C NMR spectra of raw materials and oxidized bitumen samples modified with polyethylene waste.

Sample	^1^H NMR Observations	^13^C NMR Observations	Conclusion
Heavy oil residue	Broad, unresolved peaks; dominant –CH_2_– and –CH_3_ (0.8–2.0 ppm); minor aromatics (6.5–8.0 ppm)	Intense aliphatic carbons (10–50 ppm); weak aromatic signals (120–150 ppm)	High aliphatic hydrocarbon content, slight aromatic component
Polyethylene	Sharp peaks ~1.3 ppm (–CH_2_–), ~0.9 ppm (terminal –CH_3_); no aromatics	Clear –CH_2_– (~30 ppm) and terminal carbons	Linear polyethylene
B-3-250-4	Reduced aliphatic signals; stronger aromatics; weak ~9–10 ppm (–CHO/–COOH)	Carbonyl carbons (170–200 ppm); enhanced aromatics	Moderate oxidation, partial aromatization
B-2-270-4	Weaker aliphatic signals than Sample 1; stronger aromatics and oxidized groups	More intense carbonyl and aromatic signals	Deeper oxidation, pronounced aromatization

## Data Availability

The data that supports the findings of this study are included within the manuscript. Further inquiries can be directed to the corresponding authors.
